# Supplementary material for: Lower Blood Vitamin D Levels Are Associated with Depressive Symptoms in a Population of Older Adults in Kuwait: A Cross-Sectional Study
Source: Nutrients. 2022 Apr 8;14(8):1548. doi: 10.3390/nu14081548 (PMC9032782; doi:10.3390/nu14081548)
Supplement: Supplementary file 1 [file nutrients-14-01548-s001.zip › nutrients-1633131-supplementary.pdf]

Lower blood vitamin D levels are associated with Depressive Symptoms in an Elderly Population in Kuwait: A Cross-sectional Study

Supplementary Material

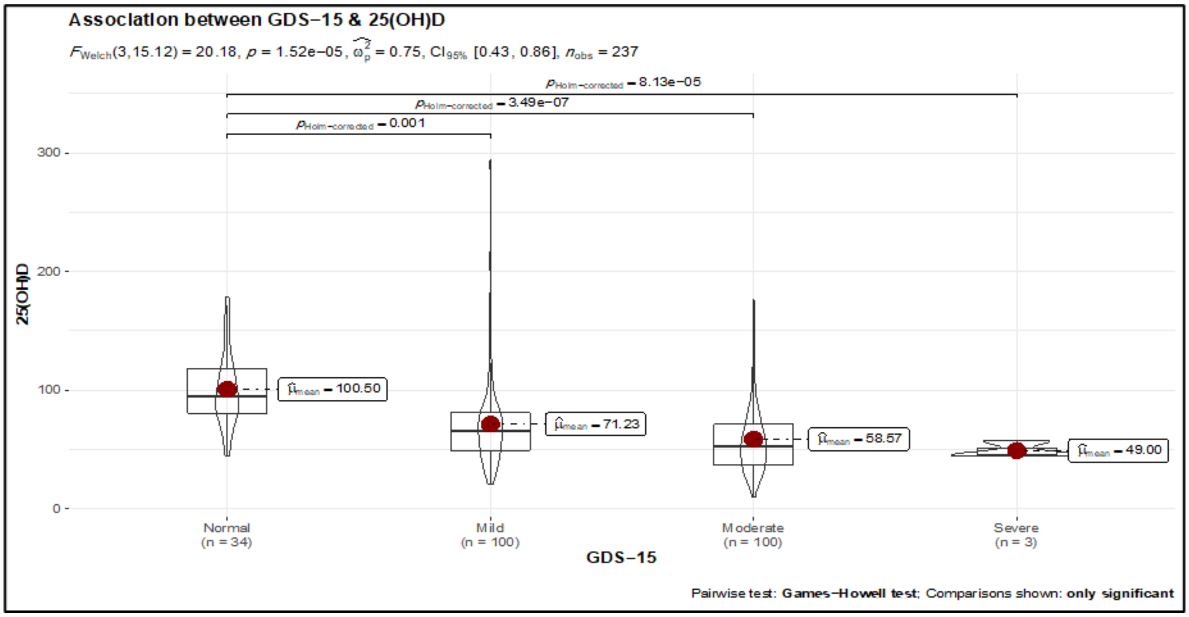

Supplementary Figure S1. Association between GDS-15 and 25(OH)D. Welch's test was statistically significant (Fwelch (3, 15.12) = 20.18, p < 0.001, $E(\omega_p^2)$ = 0.75, C.I. [0.43, 0.86]) indicating an association between GDS15 and 25-OH-D levels . Post-hoc pairwise comparisons showed that the average 25-OH-D levels were significantly higher in participants with no depression symptoms than participants with mild, moderate, and severe depressive symptoms (p < 0.001 for all pairwise comparisons).

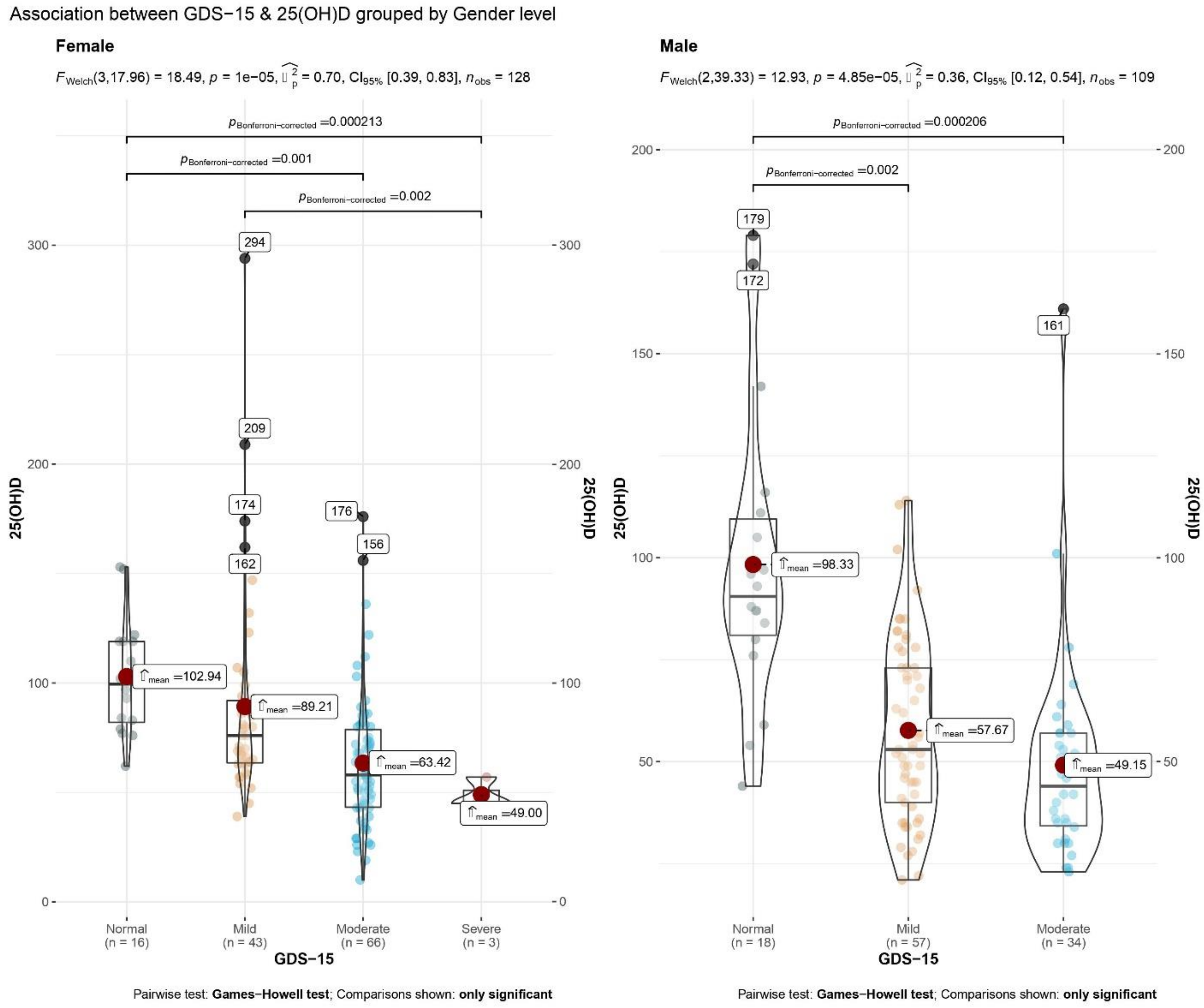

Supplementary Figure S2. Association between GDS-15 and 25-OH-D grouped by gender. When the analysis was stratified by gender, a statistically significant association was observed between GDS-15 and 25-OH-D levels in females (Fwelch (3, 17.96) = 18.49, p < 0.001, $E(\omega_p^2)$ = 0.70, C.I. [0.39, 0.83]) and males (Fwelch (2, 39.33) =12.93, p < 0.001, $\hat{\omega}_p^2$ = 0.36, C.I. [0.12, 0.54]). Pairwise comparisons indicated a statistically significant difference in the average 25-OH-D value among the pairs normal-moderate (p=0.001), normal-severe (p < 0.001) and mild – severe (p=0.002) depressive symptoms in female participants. In male participants, a significant difference was observed between the pairs normal-mild (p=0.002) and normal-moderate (p < 0.001).

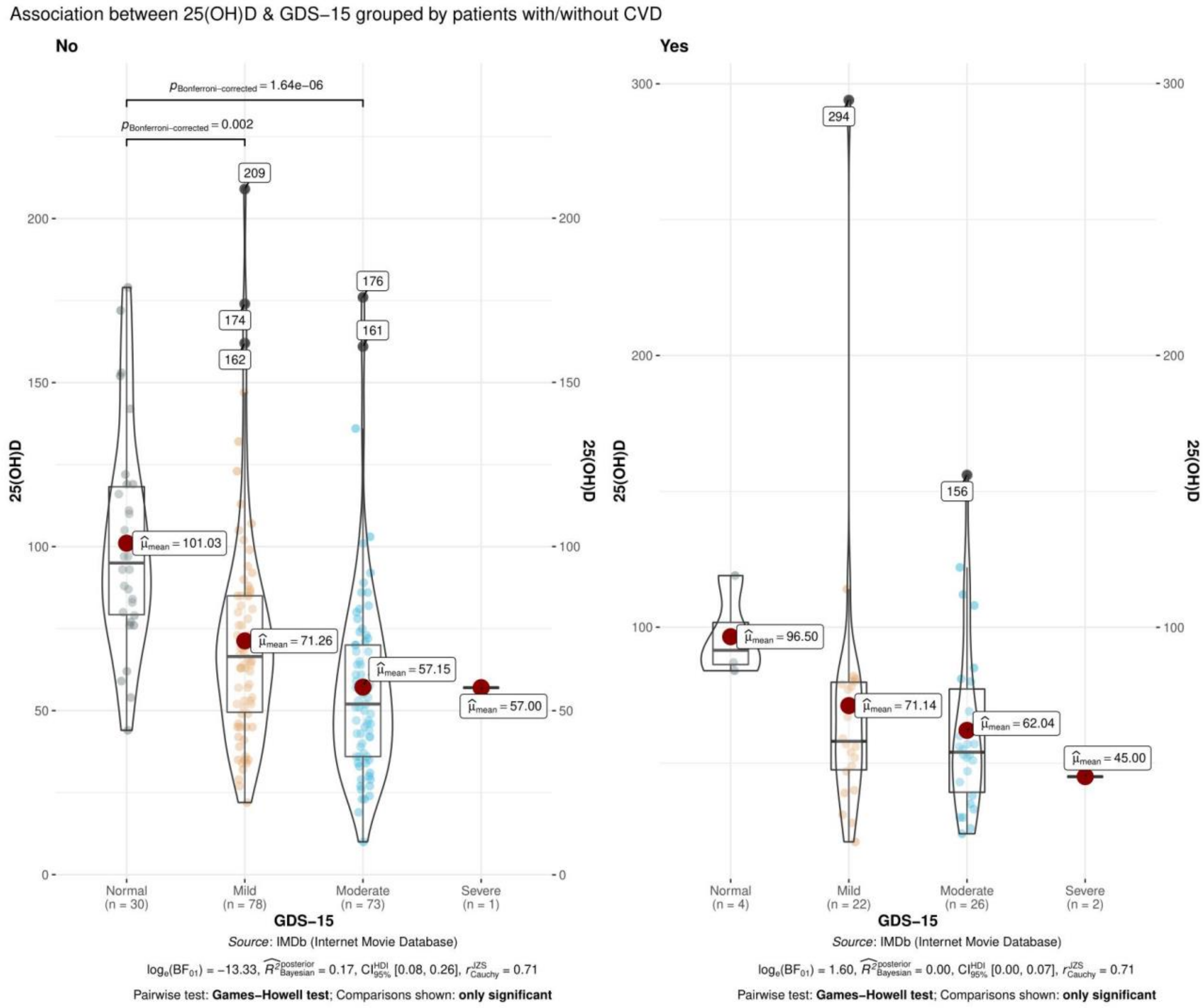

Supplementary Figure S3. Results of association between GDS-15 and 25(OH)D, grouped by CVD category. The finding shows significant association of GDS-15 with 25(OH)D, only for patients with no CVD. The Bonferroni pairwise test indicated significant differences in the average value of 25(OH)D among the pairs, normal-mild (p=0.002) and normal-moderate (p=1.64 e-06).

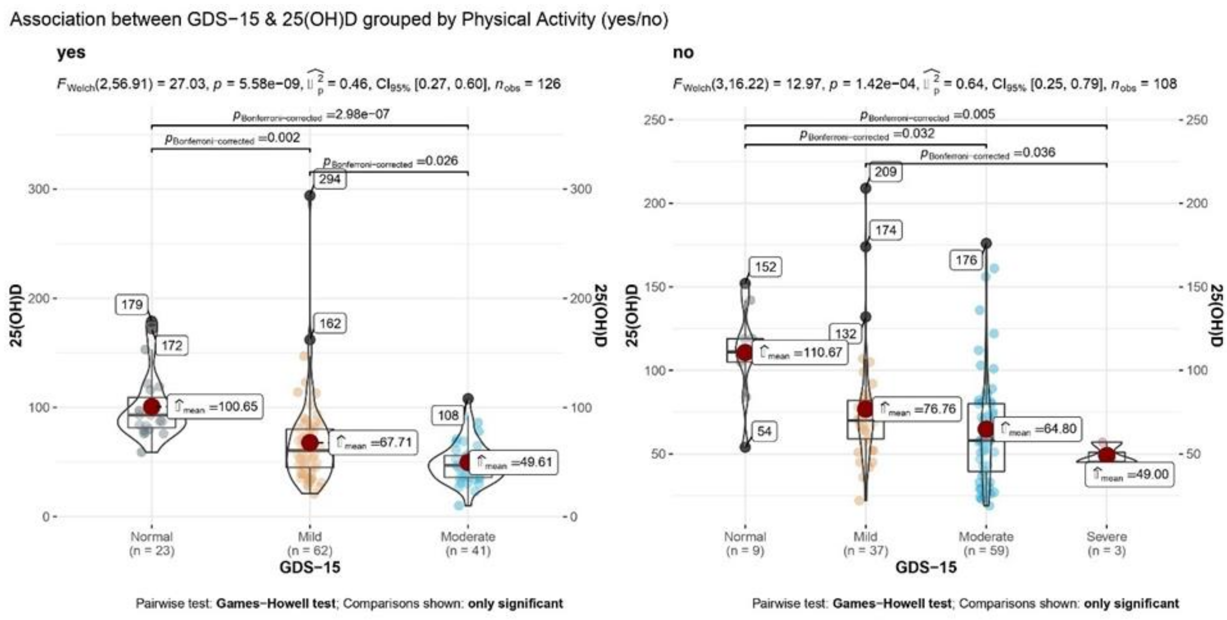

Supplementary Figure S4. Association between GDS-15 and 25(OH)D grouped by physical activity. The above finding shows significant association of GDS-15 with 25(OH)D for patients in the physical activity categories: Yes (F_{welch}(2,56.91) =27.03, p=5.58e-09, $E(\omega_p^2)$ =0.46, CI [0.27,0.60]) and No (F_{welch}(3,16.22) =12.97, p=1.42e-04, $\hat{\omega}_p^2$=0.64, CI [0.25,0.79]). For patients in no physical activity category, the Bonferroni pairwise test indicated significant difference in the average value of 25(OH)D among the pairs, normal-mild (p=0.002), normal-moderate (p=2.98e-07), and mild–moderate (p=0.026). Meanwhile, for the patients in the Yes category, the Bonferroni pairwise test indicated significant differences in the average value of 25(OH)D among the pairs, normal-moderate (p=0.032), normal-severe (p=0.005), and mild-severe (p=0.036).

Supplementary Table S1: Results of ordinal logistic regression for the association between Depression and 25(OH) D status after adjusting for other factors

Predictors	*OR*	*95% CI*	*p*
Gender [Male vs. Female]	0.34	0.14 – 0.79	**0.01**
Age	1.04	0.98 – 1.11	0.20
Marital status [Married]	Ref		
Marital status [Divorced]	1.21	0.26 –6.60	0.81
Marital status [Single]	1.78	0.19 – 39.94	0.64
Marital status [Widowed]	0.74	0.34 – 1.62	0.44
Income [<1000 KD]	Ref		
Income [1001-1500 KD]	0.73	0.31 – 1.71	0.46
Income [1501-2000 KD]	1.16	0.43 – 3.17	0.77
Income [More than 2000 KD]	1.09	0.36 – 3.38	0.88
Vitamin D supplementation [Yes vs. No]	1.06	0.36 – 3.28	0.91
Sleep duration (hours/day)	0.75	0.57 – 0.97	**0.032**
Physical activity [Yes vs. No]	0.34	0.18 – 0.63	**< 0.001**

Dyslipidemia [Yes vs. No]	1.53	0.74 – 3.15	0.25
Hypertension [Yes vs. No]	1.05	0.53 – 2.07	0.89
Cardiovascular or cerebrovascular disease [Yes vs. No]	1.94	0.98 – 3.91	0.062
Sun exposure [hours/day]	1.01	0.92 – 1.11	0.79
PTH concentration	0.95	0.86 – 1.06	0.38
Vitamin D: Deficiency	Ref		
Vitamin D: Insufficiency	6.40	2.20 – 19.91	**<0.001**
Vitamin D: Sufficiency	19.70	5.60 – 74.86	**<0.001**
OR: Odds ratio, CI: Confidence interval			
